# Reactogenicity of an Inactivated, Split-Virion Quadrivalent Influenza Vaccine in Infants and Children Aged ≥6 Months to <9 Years

**DOI:** 10.3390/vaccines13101019

**Published:** 2025-09-30

**Authors:** Terry Nolan, Frank R. Albano, Janine Oberije, Maria Piedrahita, Matthew Hohenboken

**Affiliations:** 1 Peter Doherty Institute for Infection and Immunity, University of Melbourne, Melbourne, VIC 3000, Australia; t.nolan@unimelb.edu.au; 2 Murdoch Children’s Research Institute, Melbourne, VIC 3052, Australia; 3 CSL Seqirus, Melbourne, VIC 3000, Australia; 4 CSL Seqirus, BJ 1105 Amsterdam, The Netherlands; 5 CSL Seqirus, Waltham, MA 02451, USA

**Keywords:** seasonal influenza, quadrivalent influenza vaccine, paediatric influenza vaccine formulation, infants, children

## Abstract

Background: Children are at high risk of influenza infections and may spread the disease to vulnerable family members. Quadrivalent influenza vaccines (QIV) provide protection against four strains of influenza recommended annually by the World Health Organization (WHO) and have the potential to provide improved protection during seasons with B-strain mismatch between vaccine and circulating virus strains. Methods: We evaluated the reactogenicity and safety of a QIV (Afluria Quad and Afluria Quad Junior, Seqirus, Parkville, Australia) in children aged 6 months to <3 years and 3 to <9 years over two Southern Hemisphere influenza seasons (2019 and 2020). The rates of solicited local and systemic adverse events (AEs) occurring on Days 1–7 after each vaccine dose were compared between three vaccine batches during each of the two seasons. Results: Overall, 73.7% of participants aged 6 months to <3 years and 77.5% of those aged 3 to <9 years reported any solicited AE between Day 1 and 7 of SH2019, and 66.7% and 69.2%, respectively, reported any solicited AE in SH2020, consistent with results from prior paediatric studies of QIV. The majority of solicited AEs were mild to moderate in severity. No consistent patterns of batch variation in solicited local or systemic reactogenicity were observed, suggesting no clinically significant differences between vaccine batches. No serious AEs or AEs of special interest (i.e., anaphylactic reaction or febrile convulsion) were reported during Days 1–7 after each vaccination, and no new safety concerns were identified. Conclusions: Together, these results support a clinically acceptable safety and tolerability profile of QIV in children aged 6 months to <9 years.

## 1. Introduction

Influenza is a highly infectious disease that occurs in epidemics throughout the winter in the Northern and Southern Hemispheres. Paediatric populations, particularly the youngest children, are at high risk of influenza infections, and children aged < 5 years account for nearly a million influenza-related hospitalizations worldwide [1,2]. Paediatric influenza infections also may lead to infections in older adults in the household as well cause indirect burdens on the family and society due to lost productivity from parents who must care for sick children [3,4,5,6].

Influenza vaccines are designed to protect against illness from the circulating virus strains and have historically been trivalent, including variants of A/H3N2, A/H1N1, and one B-strain lineage. Trivalent influenza vaccines (TIV) provide limited immunity against B strains of the lineage not included in the vaccine. In recent years, the circulation of the B/Yamagata strain has declined to near-undetectable levels [7]; however, some vaccine authorities continue to recommend use of quadrivalent influenza vaccines, which contain antigenic material from both type A (A/H3N2 and A/H1N1) and type B strains (B/Yamagata and B/Victoria) [4,8,9].

Relative to TIV, quadrivalent vaccines offer an improved immune response (to the B strain not included in the TIV) [10] and may improve overall vaccine protection in target populations when there are mismatches between the B strain contained within TIV and circulating virus strains [11]. B strains are major contributors to influenza morbidity and mortality, particularly in paediatric populations [11,12,13].

Starting in 2017, a split-virion, quadrivalent inactivated influenza vaccine (QIV 0.5 mL [Afluria Quad, Seqirus, Parkville, Australia]) has been licensed for use in children aged 5 years to <18 years in the USA, Australia, Canada, New Zealand, and South Korea. A split virion influenza vaccine is an inactivated influenza vaccine made by chemically disrupting the influenza virus, then purifying and splitting it into smaller pieces rather than using the whole virus particle. This process removes much of the viral genetic material and reduces reactogenicity while maintaining the key viral antigens that stimulate an immune response.

A lower dose formulation (QIV 0.25 mL [Afluria Quad Junior]) was first licensed in the USA in 2018, followed by authorizations in Australia, New Zealand, and Argentina. This study was conducted at the request of the Australian Therapeutic Goods Administration (TGA) in order to monitor the reactogenicity profile of QIV in children aged 6 months to <9 years and also to further characterize the reactogenicity profile of different manufacturing batches within the age groups 6 months to <3 years and 3 to <9 years over multiple influenza seasons.

## 2. Materials and Methods

This phase IIIb/IV study was a multicenter, randomized, open-label, uncontrolled study of QIV administered intramuscularly over Southern Hemisphere (SH) 2019 and SH 2020 influenza seasons in Australia. The study was performed to ascertain additional post-approval safety information in the population aged 6 months to less than 9 years from several manufacturing batches; therefore, no comparator vaccine was included.

After parent(s)/legal guardian(s) provided written informed consent, eligible subjects were stratified into two age cohorts: 6 months to <3 years and 3 to <9 years. Within the two age groups, subjects were randomized at the subject level in a 1:1:1 ratio to receive one of three batches of QIV per season. Participants who had not been previously vaccinated against influenza received two doses of QIV from the same batch at the dosage level indicated for their age group. Vaccination occurred on Day 1 and, for two-dose schedule subjects, on Day 29 after the first vaccination.

For 7 days after each study vaccination, parents or legal guardians were asked to record any occurrence of a prespecified set of solicited local and systemic adverse reactions and associated body temperatures in a 7-Day e-Diary. Additionally, parent(s)/legal guardian(s) of subjects were asked to contact the site if a febrile convulsion, anaphylactic reaction, or a serious adverse event (SAE) occurred. Study staff called parents/legal guardians 8 days after each study vaccination to review the e-Diary and assess the occurrence of unsolicited adverse events (AEs), including adverse events of special interest (AESIs), which included anaphylactic reaction and febrile convulsion. Findings were recorded in source documents and the clinical database.

### 2.1. Study Population

Eligible participants were male or female children aged 6 months to <9 years. An enrolment target of approximately 600 participants was planned for each season, with an approximate target of 300 participants for each of the two study age groups (6 months to <3 years and 3 to <9 years). Participants were excluded if they had a history of severe allergic reactions (e.g., anaphylaxis) to any component of the study vaccine or to a previous dose of any influenza vaccine; if they had received a licensed or investigational influenza vaccine in the 6-month period prior to enrolment; or if they had any other condition that might adversely impact participation in the study, in the opinion of the investigator.

### 2.2. Vaccine Formulation

QIV used in this study contained the four influenza strains recommended for the respective season, and the six manufacturing batches in this study were produced using the same licensed manufacturing process. Participants aged 6 months to <3 years received the 0.25 mL dose of QIV, containing at total of 30 mcg of influenza virus haemagglutinin (HA; 7.5 mcg per vaccine strain), and those aged 3 to <9 years received the 0.5 mL dose, containing 60 mcg HA (15 mcg per dose). In Season 1 (SH 2019), the vaccine strains included A/Michigan/45/2015 (H1N1) pdm09-like virus, A/Switzerland/8060/2017 (H3N2)-like virus, B/Colorado/06/2017-like virus (B/Victoria/2/87 lineage), and B/Phuket/3073/2013-like virus (B/Yamagata/16/88 lineage); in Season 2 (SH 2020), the vaccine strains were A/Brisbane/02/2018 (H1N1) pdm09-like virus, A/South Australia/34/2019 (H3N2)-like virus, B/Washington/02/2019-like virus, and B/Phuket/3073/2013-like virus.

### 2.3. Endpoints

The primary endpoint was the frequency, intensity, and duration of solicited local and systemic adverse reactions during the 7 days after each administration of QIV 0.25 mL in participants aged 6 months to <3 years or QIV 0.5 mL in participants aged 3 to <9 years. Secondary endpoints included the frequency of SAEs (including AESIs) occurring up to 7 days after the administration of QIV 0.25 mL or 0.5 mL as indicated for the age group.

### 2.4. Statistical Analyses

For the primary and secondary endpoints, the number and percentage of subjects reporting at least one solicited adverse reaction or SAE after administration of QIV were presented along with two-sided 95% confidence intervals (CI). To further characterize the solicited adverse reactions, the number and proportion of these adverse reactions were tabulated by intensity and duration over the 7 days following QIV administration.

Results were presented for each season by batch, age cohort, and study vaccine dose (first or second dose). The overall 7-day safety profile of QIV 0.25 mL or 0.5 mL across both seasons per age cohort was presented. The analyses were performed using SAS, Version 9.4.

#### Statistical Considerations for Sample Size Calculations:

With a Safety Population of 600 subjects per season, AEs with population rates of 1 in 300 have an 86.5% probability of being observed with at least one event. Events with population rates of 1 in 200 have a 95.1% chance of being observed with *n* = 600. Events with population rates of 1 in 100 have a 99.8% chance of being observed with *n* = 600 per season.

Within each cohort, a safety population of 300 subjects per season allows AEs with population rates of 1 in 300 to have a 63.3% probability of being observed with at least one event. Events with population rates of 1 in 200 have a 77.8% chance of being observed with *n* = 300. Events with population rates of 1 in 100 have a 95.1% chance of being observed with *n* = 300 per season.

Within each batch in each season, events with a population rate of 1 in 100 have a 63.4% chance of being observed with *n* = 100 (one batch per age group per season). Events with population rates of 1 in 33 have a 95.4% chance of being observed with *n* = 100.

## 3. Results

### 3.1. Subject Disposition

**Figure 1:**
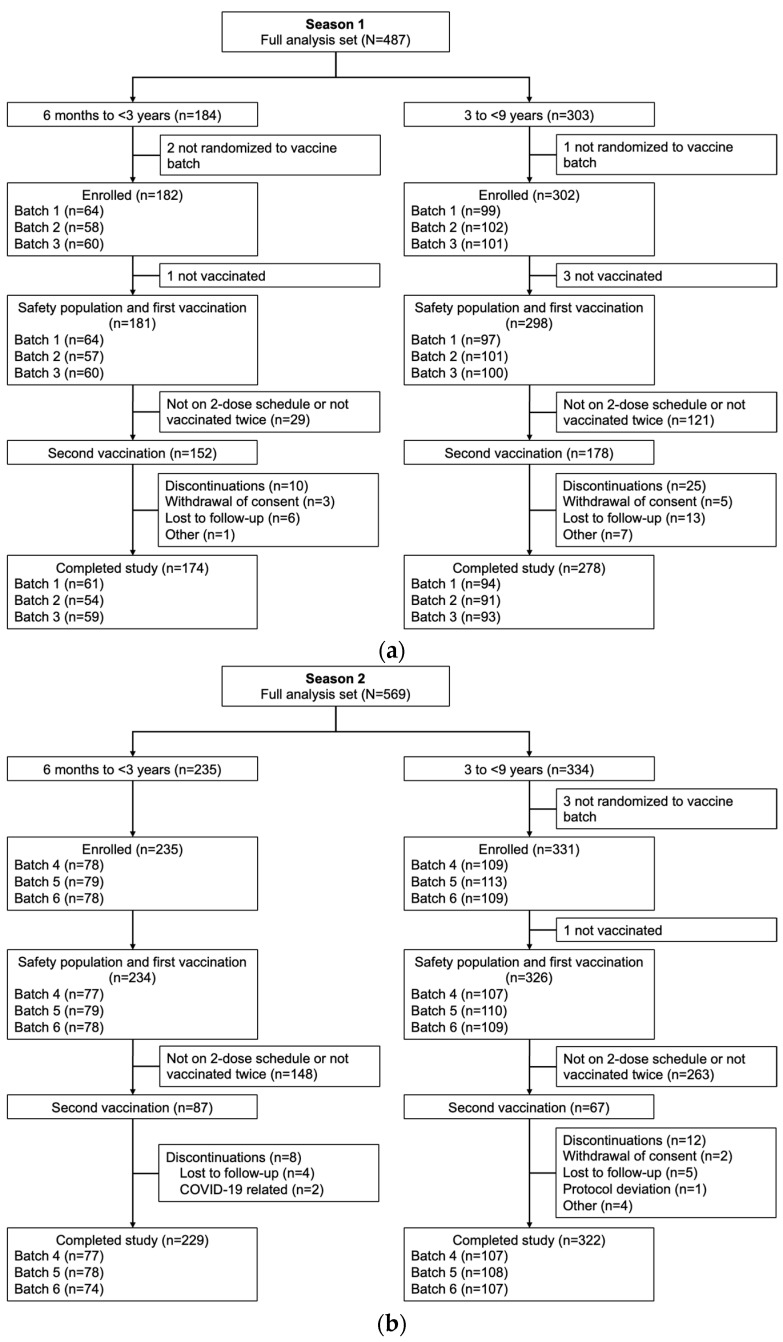
Participant disposition in Season 1 (**a**) and Season 2 (**b**).

A total of 487 children were enrolled in Season 1 (184 aged 6 months to <3 years and 303 aged 3 to <9 years), and 569 children were enrolled in Season 2 (235 aged 6 months to <3 years and 334 aged 3 to <9 years). The safety populations for each age group included 181 and 298 children, respectively, in Season 1, and 234 and 326 children in Season 2 (Figure 1).

In both seasons, the majority of participants were White or Asian. In Season 1, mean ages were 17.7 months and 5.8 years in the 6-month to <3-year and 3- to <9-year age groups, respectively. In the younger cohort, 13.3% of safety population participants had been previously vaccinated against influenza, while 34.6% of participants were previously vaccinated in the older age cohort. In Season 2, mean ages were 21.8 months and 6.0 years in the younger and older age groups, respectively, of which 60.3% and 77.6% of safety population participants had been previously vaccinated against influenza. Within each season and age group, there were no notable differences in demographic characteristics between vaccine batch groups (Table 1).

### 3.2. Reactogenicity Findings

In Season 1, any solicited adverse reactions between Day 1 and 7 were reported by 73.7% of participants aged 6 months to <3 years and 77.5% of those aged 3 to <9 years, while in Season 2, 66.7% and 69.2% of the younger and older age groups, respectively, reported a solicited adverse reaction. The proportions reporting any solicited adverse reactions across the three vaccine batches in each season were similar within each age group, although some variations were observed, likely due to the relatively small number of subjects in each batch (Table 2).

#### 3.2.1. Season 1 Reactogenicity

During Season 1, 24.0% of participants aged 6 months to <3 years experienced erythema, 22.3% injection-site pain, and 8.4% induration, with no significant differences in the rate of reactions between batches (Table S1). Among those aged 3 to <9 years, the overall rates of erythema, pain, and induration were 21.8%, 59.4%, and 11.4%, respectively, with similar proportions in each batch (Table S2). The majority of solicited local adverse reactions were of mild intensity, both for the overall population and by vaccine batch in each age group. All solicited local adverse reactions experienced in any vaccine batch had a mean onset between Day 1 and Day 2. The mean duration of all solicited local reactions was less than 2 days and was similar between vaccine batches.

Overall, the most common solicited systemic reactions in the 6-month to <3-year age group were loss of appetite, fever, and nausea and/or vomiting, which were reported by 20.1%, 18.4%, and 15.6%, respectively; reported frequencies in Batch 3 (10.0%, 13.3%, and 10.0%, respectively) were somewhat lower than rates in the other two vaccine batches (Table S1). Fever (defined as ≥37.5 °C) after any vaccination was experienced by 18.4% of subjects overall (Batch 1, 21.0% [95% CI, 11.7–33.2%]; Batch 2, 21.1% [95% CI, 11.4–33.9%]; Batch 3, 13.3% [95% CI, 5.9–24.6%]; Table S1). The rate of fever ≥38 °C was 11.7% overall. The proportion of subjects experiencing fever within the first 3 days after vaccination was 11.2% overall, 12.9% for Batch 1, 14.0% for Batch 2, and 6.8% for Batch 3.

In the 3- to <9-year age group, the most common solicited systemic reaction was malaise and fatigue (26.2% of participants overall) followed by myalgia (13.5%) and headache (12.5%), with similar frequencies in the vaccine batch groups (Table S2). The majority of solicited systemic events were mild or moderate in severity; severe events were experienced by 3.4% of participants overall and by 2.1% in Batch 1, 3.0% in Batch 2, and 5.0% in Batch 3. Severe fever was experienced by 2.3% of subjects overall (Batch 1, 2.1%; Batch 2, 2.0%; Batch 3, 3.0%); 1.3% of participants overall had a fever ≥39 °C (Table S2). Severe cases of headache (0.7%), myalgia (0.7%), and malaise and fatigue (0.7%) were also observed (Table S2).

Except for fever, most solicited systemic reactions during Season 1 occurred in the first 3 days following vaccination. Differences in the onset of solicited systemic reactions between batches were minor. The mean onset for fever was Day 4.2 overall (Batch 1, Day 4.9; Batch 2, Day 3.8; Batch 3, Day 3.9).

In both age groups, the frequency of local and systemic reactions generally decreased between the first and second vaccinations in Season 1 (Tables S1 and S2).

#### 3.2.2. Season 2 Reactogenicity

Overall, 20.5% of participants aged 6 months to <3 years subjects experienced injection-site pain in Season 2, 17.5% had erythema, and 4.3% had induration, with similar rates between batches. The majority of solicited local adverse reactions were of mild intensity, both for the overall population and by vaccine batch (Table S1). All solicited local adverse reactions experienced in any vaccine batch had a mean onset between Day 1 and Day 2. The mean duration of all solicited local reactions was less than 2 days and was similar between vaccine batches.

The most common systemic reaction in the 6-month to <3-year age group was irritability (40.2% overall). The rate of diarrhoea differed across batches (overall, 21.8%; Batch 4, 29.9%; Batch 5, 20.3%, Batch 6, 15.4%) (Table S1), whereas the frequencies of loss of appetite (15.0% overall), fever (9.4% overall), and nausea and/or vomiting (5.1% overall) were mostly consistent across all 3 batches (Table S1). Fever ≥38 °C was experienced by 4.3% of participants younger than 3 years, and fever ≥39 °C by 0.9% of subjects overall (Table S1). Most of the solicited systemic reactions occurred in the first 3 days following vaccination. The mean onset for fever was on Day 4.0 overall, Day 3.6 for Batch 4, Day 4.4 for Batch 5, and Day 3.6 for Batch 6.

In the group aged 3 to <9 years, the most common local adverse reaction was injection-site pain (53.5% overall); erythema was experienced by 18.5% and induration by 10.8%, with similar rates between batches (Table S2). The majority of events were mild to moderate in severity, and all events had a mean onset between Day 1 and Day 2 with a mean duration of <2 days, with no notable differences between vaccine batches.

Frequencies of solicited systemic reactions were similar across the three vaccine batches for the most common AEs of myalgia (12.9%), malaise and fatigue (12.0%), and headache (11.4%) (Table S2). Fever after any vaccination was experienced by 2.2% of subjects overall (Batch 4, 1.9%; Batch 5, 2.8%; Batch 6, 1.8%). The rate of fever ≥38 °C was 1.5% and of ≥39 °C was 0.6%. The proportion of subjects experiencing fever within the first 3 days after vaccination was 0.6% overall, 0.9% for Batch 4, 0% for Batch 5, and 0.9% for Batch 6. Severe cases of headache (0.3%), nausea and/or vomiting (0.6%), and malaise and fatigue (0.6%) were reported; no severe cases were observed for diarrhoea and myalgia (Table S2). Except for fever, most of the solicited systemic reactions during Season 2 occurred in the first 3 days following vaccination. The mean onset for fever was Day 4.4 overall, Day 2.5 for Batch 4, Day 6.0 for Batch 5, and Day 4.0 for Batch 6.

Similar to Season 1, the frequency of local and systemic reactions in both age groups generally decreased between the first and second vaccinations in Season 2 (Tables S1 and S2).

### 3.3. Unsolicited AEs

In Season 1, the most common related unsolicited treatment-emergent AEs were injection site bruising (6 months to <3 years: 1.7%), pyrexia (3 years to <9 years: 0.7%), and lethargy (3 years to <9 years: 0.7%). In Season 2, the most common unsolicited AEs experienced by subjects in either the younger or older group overall were teething (6 months to <3 years: 2.1%) and upper respiratory tract infection (6 months to <3 years: 2.1%; 3 years to <9 years: 0.6%) followed by rhinorrhoea (6 months to <3 years: 1.7%; 3 years to <9 years: 0.6%) (Table S3). Related unsolicited AEs were mostly reported in a single subject per age group, except for injection site pain and injection site bruising, which were reported in 2 subjects each. There were no SAEs or AESIs reported in days 1–7 after each vaccination, and no new safety concerns were identified from this study.

## 4. Discussion

In this study of QIV in children aged 6 months to <9 years, rates of solicited local and systemic adverse events were comparable with the safety findings from previous studies of QIV as well as other licensed influenza vaccines in paediatric populations [14,15,16,17,18,19]. The frequency and intensity of solicited local and systemic adverse reactions were consistent between the two study seasons, and the event rates within age subgroups were as expected from previous clinical studies. In both seasons, local adverse reactions were more commonly reported by subjects in the ≥3- to 9-year age group, whereas systemic adverse reactions were more common in the ≥6-month to <3-year age group. No SAEs or AESIs were reported during Days 1–7 after each vaccination, and no new safety concerns were identified.

In this study, we compared rates of AEs across three different batches in each season. In general, both solicited local and systemic reactogenicity did not suggest any pattern of variation between the numerous clinical batches. Thus, we found no evidence of clinically significant differences between vaccine batches. Numerically, there were some batch variations for some adverse reactions, but these were frequently associated with relatively small group sizes for subgroup analyses.

Globally, children younger than 5 years are hospitalized for influenza at higher rates than those of any other age group (including older adults) [20,21]. Differences between the immune systems of adults and those of young children appear to account for children’s vulnerability to influenza infection and severe disease. Possible mechanisms include impaired viral clearance, excessive inflammatory responses to infection, and compensatory anti-inflammatory responses that increase susceptibility to pneumonia and other secondary infections [22]. Influenza-related illness in children is associated with high direct medical costs related to both outpatient, emergency, and inpatient care, and indirect costs due to lost productivity from adult caregivers [3,4,5,6,23]. Paediatric vaccination not only reduces the burden of influenza disease in children, it may reduce viral transmission in the community, preventing exposure to vulnerable older household members [24]. Finally paediatric vaccination has been shown to be cost-effective [23]. For these reasons, annual seasonal influenza vaccination for young children is considered a priority throughout the world [4,25,26].

Limitations of this study include relatively small sample sizes in the batch and age subgroups, which limit statistical analyses. The second season, in 2020, occurred during the COVID-19 pandemic, when there may have been unknown/undetected infections with SARS-CoV-2. Any possible impact of the pandemic cannot be discerned from the data from the present study. However, unrecognized SARS-CoV-2 infections may have influenced reported rates of adverse reactions. In addition, the social atmosphere may have subtly altered parents’ observations during that season. It should also be noted that adverse reaction reporting in the younger age subgroup was based on parental observations, whereas reactions in older subjects were self-reported.

Overall, our findings support the safety of influenza vaccination for children aged <9 years. This is consistent with health authority recommendations that children be immunized against influenza. Young children, who have limited antigenic exposure, are at greater risk than older children and adults <65 years for severe infection [4,27,28]. In addition, influenza transmission from school-age children to other household members may pose a risk to older adults or those who are immunocompromised [3,4,5,6].

## 5. Conclusions

This study demonstrated that QIV 0.5 mL and QIV 0.25 mL continue to have clinically acceptable safety and tolerability profiles in children aged 6 months to <9 years.

## Figures and Tables

**Table 1:** Demographics and characteristics of study participants at baseline.

Season 1, SH 2019		6 Months to <3 Years				3 to <9 Years			
		Batch 1 (*n* = 64)	Batch 2 (*n* = 58)	Batch 3 (*n* = 60)	Total (*n* = 184)	Batch 1 (*n* = 99)	Batch 2 (*n* = 102)	Batch 3 (*n* = 101)	Total (*n* = 303)
Age, mean (SD)		17.7 (9.12)	17.1 (9.28)	18.3 (10.06)	17.7 (9.40)	66.5 (20.19)	73.3 (19.70)	70.7 (20.36)	70.1 (20.21)
Female sex, *n* (%)		30 (46.9)	29 (50.0)	30 (50.0)	91 (49.5)	45 (45.5)	50 (49.0)	55 (54.5)	151 (49.8)
Race and ethnicity	American Indian/Alaska Native	1 (1.6)	0	0	1 (0.5)	0	0	0	0
	Asian	13 (20.3)	11 (19.0)	11 (18.3)	36 (19.6)	11 (11.1)	5 (4.9)	10 (9.9)	26 (8.6)
	Black or African American	1 (1.6)	0	0	1 (0.5)	0	0	0	0
	Native Hawaiian or other Pacific Islander	0	0	0	0	2 (2.0)	3 (2.9)	2 (2.0)	7 (2.3)
	White	41 (64.1)	43 (74.1)	45 (75.0)	130 (70.7)	79 (79.8)	87 (85.3)	83 (82.2)	250 (82.5)
	Other ^a^	8 (12.5)	4 (6.9)	4 (6.7)	16 (8.7)	7 (7.1)	7 (6.9)	6 (5.9)	20 (6.6)
	Hispanic ethnicity	2 (3.1)	0	1 (1.7)	3 (1.6)	2 (2.0)	1 (1.0)	3 (3.0)	6 (2.0)
Previous influenza vaccination ^b^		5/64 (7.8)	6/57 (10.5)	13/60 (21.7)	24/181 (13.3)	32/97 (33.0)	40/101 (39.6)	31/100 (31.0)	103/298 (34.6)
**Season 2, SH 2020**		**Batch 4** **(*n* = 78)**	**Batch 5** **(*n* = 79)**	**Batch 6** **(*n* = 78)**	**Total** **(*n* = 235)**	**Batch 4** **(*n* = 109)**	**Batch 5** **(*n* = 113)**	**Batch 6** **(*n* = 109)**	**Total** **(*n* = 334)**
Age, mean (SD)		20.5 (8.09)	22.3 (8.15)	22.5 (8.10)	21.8 (8.13)	69.9 (19.87)	69.8 (19.82)	74.5 (19.16)	71.4 (19.69)
Female sex, *n* (%)		43 (55.1)	36 (45.6)	40 (51.3)	119 (50.6)	42 (38.5)	53 (46.9)	60 (55.0)	156 (46.7)
Race and ethnicity	American Indian/Alaska Native	0	0	1 (1.3)	1 (0.4)	0	0	0	0
	Asian	9 (11.5)	11 (13.9)	10 (12.8)	30 (12.8)	9 (8.3)	15 (13.3)	10 (9.2)	35 (10.5)
	Black or African American	0	0	0	0	0	0	0	0
	Native Hawaiian or other Pacific Islander	1 (1.3)	0	3 (3.8)	4 (1.7)	2 (1.8)	0	0	2 (0.6)
	White	59 (75.6)	64 (81.0)	55 (70.5)	178 (75.7)	94 (86.2)	93 (82.3)	93 (85.3)	282 (84.4)
	Other ^a^	9 (11.5)	4 (5.1)	9 (11.5)	22 (9.4)	4 (3.7)	5 (4.4)	6 (5.5)	15 (4.5)
	Hispanic ethnicity	1 (1.3)	1 (1.3)	3 (3.8)	5 (2.1)	3 (2.8)	0	4 (3.7)	7 (2.1)
Previous influenza vaccination ^b^		47/77 (61.0)	48/79 (60.8)	46/78 (59.0)	141/234 (60.3)	85/107 (79.4)	86/109 (79.0)	82/110 (74.5)	103/326 (77.6)

SD, standard deviation; SH, Southern Hemisphere. ^a^ Includes subjects of indigenous ancestry, including Aboriginal, Torres Strait Islander, or Māori. ^b^ Based on safety population.

**Table 2:** Incidence of solicited adverse reactions and unsolicited AEs over two influenza seasons.

Season 1, SH 2019		6 Months to <3 Years				3 to <9 Years			
		Batch 1 (*n* = 62)	Batch 2 (*n* = 57)	Batch 3 (*n* = 60)	Total (*n* = 179)	Batch 1 (*n* = 97)	Batch 2 (*n* = 101)	Batch 3 (*n* = 100)	Total (*n* = 298)
Solicited adverse reactions	Any	50 (80.6)	41 (71.9)	41 (68.3)	132 (73.7)	72 (74.2)	74 (73.3)	85 (85.0)	231 (77.5)
	Any severe	7 (11.3)	2 (3.5)	5 (8.3)	14 (7.8)	6 (6.2)	8 (7.9)	9 (9.0)	23 (7.7)
	Local	26 (41.9)	27 (47.4)	14 (23.3)	67 (37.4))	59 (60.8)	64 (63.4)	70 (70.0)	193 (64.8)
	Severe local	0	0	0	0	4 (4.1)	5 (5.0)	5 (5.0)	14 (4.7)
	Systemic	41 (66.1)	33 (57.9)	37 (61.7)	111 (62.0)	47 (48.5)	39 (38.6)	52 (52.0)	138 (46.3)
	Severe systemic	7 (11.3)	2 (3.5)	5 (8.3)	14 (7.8)	2 (2.1)	3 (3.0)	5 (5.0)	10 (3.4)
Unsolicited AEs	Any	11 (17.2)	8 (14.0)	14 (23.3)	33 (18.2)	14 (14.4)	6 (5.9)	12 (12.0)	32 (10.7)
	Severe	1 (1.6)	1 (1.8)	0	2 (1.1)	0	0	2 (2.0)	2 (0.7)
	Related	1 (1.6)	1 (1.8)	2 (3.3)	4 (2.2)	6 (6.2)	1 (1.0)	1 (1.0)	8 (2.7)
	SAEs	0	0	0	0	0	0	0	0
	AE leading to withdrawal	0	0	0	0	0	0	0	0
	AESIs	0	0	0	0	0	0	0	0
	Deaths	0	0	0	0	0	0	0	0
**Season 2, SH 2020**		**Batch 4** **(*n* = 77)**	**Batch 5** **(*n* = 79)**	**Batch 6** **(*n* = 78)**	**Total** **(*n* = 234)**	**Batch 4** **(*n* = 107)**	**Batch 5** **(*n* = 109)**	**Batch 6** **(*n* = 109)**	**Total** **(*n* = 325)**
Solicited adverse reactions	Any	48 (62.3)	57 (72.2)	51 (65.4)	156 (66.7)	73 (68.2)	72 (66.1)	80 (73.4)	225 (69.2)
	Any severe	2 (2.6)	3 (3.8)	4 (5.1)	9 (3.8)	5 (4.7)	1 (0.9)	6 (5.5)	12 (3.7)
	Local	19 (24.7)	30 (38.0)	28 (35.9)	77 (32.9)	65 (60.7)	65 (59.6)	72 (66.1)	202 (62.2)
	Severe local	1 (1.3)	0	0	1 (0.4)	2 (1.9)	1 (0.9)	4 (3.7)	7 (2.2)
	Systemic	42 (54.5)	47 (59.5)	38 (48.7)	127 (54.3)	33 (30.8)	33 (30.3)	36 (33.0)	102 (31.4)
	Severe systemic	1 (1.3)	3 (3.8)	4 (5.1)	8 (3.4)	3 (2.8)	0	2 (1.8)	5 (1.5)
Unsolicited AEs	Any	10 (13.0)	11 (13.9)	13 (16.7)	34 (14.5)	5 (4.7)	9 (8.2)	5 (4.6)	19 (5.8)
	Severe	0	1 (1.3)	2 (2.6)	3 (1.3)	0	1 (0.9)	1 (0.9)	2 (0.6)
	Related	2 (2.6)	2 (2.5)	2 (2.6)	6 (2.6)	0	4 (3.6)	1 (0.9)	5 (1.5)
	SAEs	0	0	0	0	0	0	0	0
	AE leading to withdrawal	0	0	0	0	0	0	0	0
	AESIs	0	0	0	0	0	0	0	0
	Deaths	0	0	0	0	0	0	0	0

AE, adverse event; AESI, adverse event of special interest; SAE, serious adverse event; SH, Southern Hemisphere.

## Data Availability

Data are contained within the article and supplementary materials.

## Supplementary Materials

The following supporting information can be downloaded at: https://www.mdpi.com/article/10.3390/vaccines13101019/s1, Table S1: Solicited local and systemic adverse reactions experienced after any vaccination and after the first and second vaccinations in participants aged 6 months to <3 years, Day 1–7 (solicited safety population); Table S2: Solicited local and systemic adverse reactions experienced after any vaccination and after the first and second vaccinations in participants aged 3 to <9 years, Day 1–7 (solicited safety population); Table S3: Unsolicited AEs occurring in ≥1% of participants by system organ class and preferred term after any vaccination, Day 1–7 (safety population).
