# Supplementary material for: Reactogenicity of an Inactivated, Split-Virion Quadrivalent Influenza Vaccine in Infants and Children Aged ≥6 Months to <9 Years
Source: Vaccines (Basel). 2025 Sep 30;13(10):1019. doi: 10.3390/vaccines13101019 (PMC12568138; doi:10.3390/vaccines13101019)
Supplement: Supplementary file 1 [file vaccines-13-01019-s001.zip › vaccines-3862914-supplementary.pdf]

Supplementary Materials for:

Reactogenicity of an inactivated, split-virion quadrivalent influenza vaccine in infants and children aged ≥6 months to <9 years

Terry Nolan, Frank R. Albano, Janine Oberije, Maria Piedrahita, and Matthew Hohenboken

Table S1. Solicited local and systemic adverse reactions experienced after any vaccination and after the first and second vaccinations in participants aged 6 months to <3 years, Day 1–7 (solicited safety population)

	**After any vaccination**				**After first vaccination**				**After second vaccination**			
**Season 1**	**Batch 1 (n=62)**	**Batch 2 (n=57)**	**Batch 3 (n=60)**	**Total (n=179)**	**Batch 1 (n=62)**	**Batch 2 (n=55)**	**Batch 3 (n=60)**	**Total (n=177)**	**Batch 1 (n=55)**	**Batch 2 (n=47)**	**Batch 3 (n=43)**	**Total (n=145)**
**Local reactions, n (%)**												
Pain at injection site, any, n (%) (95% CI)[a]	14 (22.6) (12.9, 35.0)	16 (28.1) (17.0, 41.5)	10 (16.7) (8.3, 28.5)	40 (22.3) (16.5, 29.2)	11 (17.7) (9.2, 29.5)	15 (27.3) (16.1, 41.0)	10 (16.7) (8.3, 28.5)	36 (20.3) (14.7, 27.0)	6 (10.9) (4.1, 22.2)	7 (14.9) (6.2, 28.3)	3 (7.0) (1.5, 19.1)	16 (11.0) (6.4, 17.3)
Mild (grade 1)	12 (19.4)	12 (21.1)	10 (16.7)	34 (19.0)	10 (16.1)	11 (20.0)	10 (16.7)	31 (17.5)	5 (9.1)	7 (14.9)	3 (7.0)	15 (10.3)
Moderate (grade 2)	2 (3.2)	4 (7.0)	0	6 (3.4)	1 (1.6)	4 (7.3)	0	5 (2.8)	1 (1.8)	0	0	1 (0.7)
Severe (grade 3)	0	0	0	0	0	0	0	0	0	0	0	0
Erythema at injection site, any, n (%) (95% CI)[b]	20 (32.3) (20.9, 45.3)	14 (24.6) (14.1, 37.8)	9 (15.0) (7.1, 26.6)	43 (24.0) (18.0, 31.0)	15 (24.2) (14.2, 36.7)	14 (25.5) (14.7, 39.0)	6 (10.0) (3.8, 20.5)	35 (19.8) (14.2, 26.4)	8 (14.5) (6.5, 26.7)	3 (6.4) (1.3, 17.5)	5 (11.6) (3.9, 25.1)	16 (11.0) (6.4, 17.3)
Mild (grade 1)	16 (25.8)	10 (17.5)	6 (10.0)	32 (17.9)	14 (22.6)	11 (20.0)	4 (6.7)	29 (16.4)	5 (9.1)	2 (4.3)	4 (9.3)	11 (7.6)
Moderate (grade 2)	4 (6.5)	4 (7.0)	3 (5.0)	11 (6.1)	1 (1.6)	3 (5.5)	2 (3.3)	6 (3.4)	3 (5.5)	1 (2.1)	1 (2.3)	5 (3.4)
Severe (grade 3)	0	0	0	0	0	0	0	0	0	0	0	0
Induration/swelling at injection site, any n (%) (95% CI)[b]	7 (11.3) (4.7, 21.9)	4 (7.0) (1.9, 17.0)	4 (6.7) (1.8, 16.2)	15 (8.4) (4.8, 13.4)	5 (8.1) (2.7, 17.8)	3 (5.5) (1.1, 15.1)	4(6.7) (1.8, 16.2)	12(6.8) (3.6, 11.5)	3(5.5) (1.1, 15.1)	2(4.3) (0.5, 14.5)	0 (0.0, 8.2)	5 (3.4) (1.1, 7.9)
Mild (grade 1)	5 (8.1)	1 (1.8)	2 (3.3)	8 (4.5)	4 (6.5)	1 (1.8)	2 (3.3)	7 (4.0)	2 (3.6)	1 (2.1)	0	3 (2.1)
Moderate (grade 2)	2 (3.2)	3 (5.3)	2 (3.3)	7 (3.9)	1 (1.6)	2 (3.6)	2 (3.3)	5 (2.8)	1 (1.8)	1 (2.1)	0	2 (1.4)
Severe (grade 3)	0	0	0	0	0	0	0	0	0	0	0	0
**Systemic reactions, n (%)**												
Fever, any, n (%) (95% CI)[c]	13 (21.0) (11.7, 33.2)	12 (21.1) (11.4, 33.9)	8 (13.3) (5.9, 24.6)	33 (18.4) (13.0, 24.9)	9 (14.5) (6.9, 25.8)	6 (10.9) (4.1, 22.2)	5 (8.3) (2.8, 18.4)	20 (11.3) (7.0, 16.9)	7 (12.7) (5.3, 24.5)	7 (14.9) (6.2, 28.3)	3 (7.0) (1.5, 19.1)	17 (11.7) (7.0, 18.1)
Mild (grade 1)	4 (6.5)	6 (10.5)	2 (3.3)	12 (5.1)	4 (6.5)	3 (5.5)	2 (3.3) 2	9 (5.1)	2 (3.6)	4 (8.5)	0	6 (4.1)
Moderate (grade 2)	3 (4.8)	4 (7.0)	1 (1.7)	3 (1.3)	2 (3.2)	1 (1.8)	0	3 (1.7)	2 (3.6)	3 (6.4)	1 (2.3)	6 (4.1)
Severe (grade 3)	6 (9.7)	2 (3.5)	5 (8.3)	7 (3.0)	3 (4.8)	2 (3.6)	3 (5.0)	8 (4.5)	3 (5.5)	0	2 (4.7)	5 (3.4)
Fever ≥38°C	9 (14.5)	6 (10.5)	6 (10.0)	10 (4.3)	5 (8.1)	3 (5.5)	3 (5.0)	11 (6.2)	5 (9.1)	3 (6.4)	3 (7.0)	11 (7.6)
Fever ≥39°C	2 (3.2)	1 (1.8)	1 (1.7)	2 (0.9)	1 (1.6)	1 (1.8)	1 (1.7)	3 (1.7)	1 (1.8)	0	0	1 (0.7)
Diarrhoea, any, n (%) (95% CI)[d]	21 (33.9) (22.3, 47.0)	23 (40.4) (27.6, 54.2)	20 (33.3) (21.7,46.7)	51 (21.8) (16.7, 27.6)	15 (24.2) (14.2, 36.7)	16 (29.1) (17.6, 42.9)	15 (25.0) (14.7, 37.9)	46 (26.0) (19.7, 33.1)	9 (16.4) (7.8, 28.8)	13 (27.7) (15.6, 42.6)	11 (25.6) (13.5, 41.2)	33 (22.8) (16.2, 30.5)
Mild (grade 1)	20 (32.3)	17 (29.8)	14 (23.3)	46 (19.7)	14 (22.6)	12 (21.8)	14 (23.3)	40 (22.6)	9 (16.4)	11 (23.4)	6 (14.0)	26 (17.9)
Moderate (grade 2)	1 (1.6)	5 (8.8)	6 (10.0)	5 (2.1)	1 (1.6)	3 (5.5)	1 (1.7)	5 (2.8)	0	2 (4.3)	5 (11.6)	7 (4.8)

	After any vaccination				After first vaccination				After second vaccination			
**Season 1**	**Batch 1 (n=62)**	**Batch 2 (n=57)**	**Batch 3 (n=60)**	**Total (n=179)**	**Batch 1 (n=62)**	**Batch 2 (n=55)**	**Batch 3 (n=60)**	**Total (n=177)**	**Batch 1 (n=55)**	**Batch 2 (n=47)**	**Batch 3 (n=43)**	**Total (n=145)**
Severe (grade 3)	0	1 (1.8)	0	0	0	1 (1.8)	0	1 (0.6)	0	0	0	0
Nausea and/or vomiting, any, n (%) (95% CI)[d]	10 (16.1) (8.0, 27.7)	12 (21.1) (11.4, 33.9)	6 (10.0) (3.8, 20.5)	12 (5.1) (2.7, 8.8)	8 (12.9) (5.7, 23.9)	7 (12.7) (5.3, 24.5)	5 (8.3) (2.8, 18.4)	20 (11.3) (7.0, 16.9)	2 (3.6) (0.4, 12.5)	6 (12.8) (4.8, 25.7)	1 (2.3) (0.1, 12.3)	9 (6.2) (2.9, 11.5)
Mild (grade 1)	7 (11.3)	7 (12.3)	3 (5.0)	8 (3.4)	6 (9.7)	4 (7.3)	3 (5.0)	13 (7.3)	1 (1.8)	4 (8.5)	0	5 (3.4)
Moderate (grade 2)	3 (4.8)	4 (7.0)	3 (5.0)	3 (1.3)	2 (3.2)	2 (3.6)	2 (3.3)	6 (3.4)	1 (1.8)	2 (4.3)	1 (2.3)	4 (2.8)
Severe (grade 3)	0	1 (1.8)	0	1 (0.4)	0	1 (1.8)	0	1 (0.6)	0	0	0	0
Loss of appetite, any, n (%) (95% CI)[d]	14 (22.6) (12.9, 35.0)	16 (28.1) (17.0, 41.5)	6 (10.0) (3.8, 20.5)	36 (20.1) (14.5, 26.7)	10 (16.1) (8.0, 27.7)	9 (16.4) (7.8, 28.8)	5 (8.3) (2.8, 18.4)	24 (13.6) (8.9, 19.5)	8 (14.5) (6.5, 26.7)	8 (17.0) (7.6, 30.8)	2 (4.7) (0.6, 15.8)	18 (12.4) (7.5, 18.9)
Mild (grade 1)	6 (9.7)	11 (19.3)	3 (5.0)	20 (11.2)	5 (8.1)	5 (9.1)	2 (3.3)	12 (6.8)	4 (7.3)	6 (12.8)	1 (2.3)	11 (7.6)
Moderate (grade 2)	7 (11.3)	4 (7.0)	3 (5.0)	14 (7.8)	4 (6.5)	3 (5.5)	3 (5.0)	10 (5.6)	4 (7.3)	2 (4.3)	1 (2.3)	7 (4.8)
Severe (grade 3)	1 (1.6)	1 (1.8)	0	2 (1.1)	1 (1.6)	1 (1.8)	0	2 (1.1)	0	0	0	0
Irritability, any, n (%) (95% CI)[d]	32 (51.6) (38.6, 64.5)	27 (47.4) (34.0, 61.0)	27 (45.0) (32.1, 58.4)	86 (48.0) (40.5, 55.6)	20 (32.3) (20.9, 45.3)	22 (40.0) (27.0, 54.1)	21 (35.0) (23.1, 48.4)	63 (35.6) (28.6, 43.1)	21 (38.2) (25.4, 52.3)	13 (27.7) (15.6, 42.6)	12 (27.9) (15.3, 43.7)	46 (31.7) (24.3, 40.0)
Mild (grade 1)	21 (33.9)	12 (21.1)	14 (23.3)	47 (26.3)	13 (21.0)	10 (18.2)	10 (16.7)	33 (18.6)	14 (25.5)	10 (21.3)	6 (14.0)	30 (20.7)
Moderate (grade 2)	9 (14.5)	14 (24.6)	12 (20.0)	35 (19.6)	6 (9.7)	11 (20.0)	10 (16.7)	27 (15.3)	6 (10.9)	3 (6.4)	5 (11.6)	14 (9.7)
Severe (grade 3)	2 (3.2)	1 (1.8)	1 (1.7)	4 (2.2)	1 (1.6)	1 (1.8)	1 (1.7)	3 (1.7)	1 (1.8)	0	1 (2.3)	2 (1.4)

	After any vaccination				After first vaccination				After second vaccination			
**Season 2**	**Batch 4 (n=77)**	**Batch 5 (n=79)**	**Batch 6 (n=78)**	**Total (n=234)**	**Batch 4 (n=77)**	**Batch 5 (n=79)**	**Batch 6 (n=78)**	**Total (n=234)**	**Batch 4 (n=29)**	**Batch 5 (n=30)**	**Batch 6 (n=28)**	**Total (n=87)**
**Local reactions, n (%)**												
Pain at injection site, any, n (%) (95% CI)[a]	10 (13.0) (6.4, 22.6)	17 (21.5) (13.1, 32.2)	21 (26.9) (17.5, 38.2)	48 (20.5) (15.5, 26.3)	10 (13.0) (6.4, 22.6)	17 (21.5) (13.1, 32.2)	20 (25.6) (16.4, 36.8)	47 (20.1) (15.1, 25.8)	1 (3.4) (0.1, 17.8)	1 (3.3) (0.1, 17.2)	2 (7.1) (0.9, 23.5)	4 (4.6) (1.3, 11.4)
Mild (grade 1)	8 (10.4)	10 (12.7)	19 (24.4)	37 (15.8)	8 (10.4)	10 (12.7)	18 (23.1)	36 (15.4)	1 (3.4)	1 (3.3)	2 (7.1)	4 (4.6)
Moderate (grade 2)	2 (2.6)	7 (8.9)	2 (2.6)	11 (4.7)	2 (2.6)	7 (8.9)	2 (2.6)	11 (4.7)	0	0	0	0
Severe (grade 3)	0	0	0	0	0	0	0	0	0	0	0	0
Erythema at injection site, any, n (%) (95% CI)[b]	13 (16.9) (9.3, 27.1)	18 (22.8) (14.1, 33.6)	10 (12.8) (6.3, 22.3)	41 (17.5) (12.9, 23.0)	13 (16.9) (9.3, 27.1)	17 (21.5) (13.1, 32.2)	9 (11.5) (5.4, 20.8)	39 (16.7) (12.1, 22.1)	2 (6.9) (0.8, 22.8)	3 (10.0) (2.1, 26.5)	1 (3.6) (0.1, 18.3)	6 (6.9) (2.6, 14.4)
Mild (grade 1)	7 (9.1)	16 (20.3)	6 (7.7)	29 (12.4)	7 (9.1)	15 (19.0)	5 (6.4)	27 (11.5)	2 (6.9)	3 (10.0)	1 (3.6)	6 (6.9)
Moderate (grade 2)	5 (6.5)	2 (2.5)	4 (5.1)	11 (4.7)	5 (6.5)	2 (2.5)	4 (5.1)	11 (4.7)	0	0	0	0

	After any vaccination				After first vaccination				After second vaccination			
**Season 2**	**Batch 4 (n=77)**	**Batch 5 (n=79)**	**Batch 6 (n=78)**	**Total (n=234)**	**Batch 4 (n=77)**	**Batch 5 (n=79)**	**Batch 6 (n=78)**	**Total (n=234)**	**Batch 4 (n=29)**	**Batch 5 (n=30)**	**Batch 6 (n=28)**	**Total (n=87)**
Severe (grade 3)	1 (1.3)	0	0	1 (0.4)	1 (1.3)	0	0	1 (0.4)	0	0	0	0
Induration/swelling at injection site, any n (%) (95% CI)[b]	3 (3.9) (0.8, 11.0)	3 (3.8) (0.8, 10.7)	4 (5.1) (1.4, 12.6)	10 (4.3) (2.1, 7.7)	3 (3.9) (0.8, 11.0)	3 (3.8) (0.8, 10.7)	3 (3.8) (0.8, 10.8)	9 (3.8) (1.8, 7.2)	0 (0.0, 11.9)	0 (0.0, 11.6)	1 (3.6) (0.1, 18.3)	1 (1.1) (0.0, 6.2)
Mild (grade 1)	0	2 (2.5)	4 (5.1)	6 (2.6)	0	2 (2.5)	3 (3.8)	5 (2.1)	0	0	1 (3.6)	1 (1.1)
Moderate (grade 2)	3 (3.9)	1 (1.3)	0	4 (1.7)	3 (3.9)	1 (1.3)	0	4 (1.7)	0	0	0	0
Severe (grade 3)	0	0	0	0	0	0	0	0	0	0	0	0
**Systemic reactions, n (%)**												
Fever, any, n (%) (95% CI)[c]	7 (9.1) (3.7, 17.8)	10 (12.7) (6.2, 22.0)	5 (6.4) (2.1, 14.3)	22 (9.4) (6.0, 13.9)	5 (6.5) (2.1, 14.5)	9 (11.4) (5.3, 20.5)	3 (3.8) (0.8, 10.8)	17 (7.3) (4.3, 11.4)	4 (13.8) (3.9, 31.7)	3 (10.0) (2.1, 26.5)	4 (14.3) (4.0, 32.7)	11 (12.6) (6.5, 21.5)
Mild (grade 1)	4 (5.2)	6 (7.6)	2 (2.6)	12 (5.1)	4 (5.2)	5 (6.3)	3 (3.8)	12 (5.1)	2 (6.9)	2 (6.7)	1 (3.6)	5 (5.7)
Moderate (grade 2)	2 (2.6)	1 (1.3)	0	3 (1.3)	1 (1.3)	1 (1.3)	0	2 (0.9)	1 (3.4)	1 (3.3)	0	2 (2.3)
Severe (grade 3)	1 (1.3)	3 (3.8)	3 (3.8)	7 (3.0)	0	3 (3.8)	0	3 (1.3)	1 (3.4)	0	3 (10.7)	4 (4.6)
Fever ≥38°C	3 (3.9)	4 (5.1)	3 (3.8)	10 (4.3)	1 (1.3)	4 (5.1)	0	5 (2.1)	2 (6.9)	1 (3.3)	3 (10.7)	6 (6.9)
Fever ≥39°C	0	2 (2.5)	0	2 (0.9)	0	2 (2.5)	0	2 (0.9)	0	0	0	0
Diarrhoea, any, n (%) (95% CI)[d]	23 (29.9) (20.0, 41.4)	16 (20.3) (12.0, 30.8)	12 (15.4) (8.2, 25.3)	51 (21.8) (16.7, 27.6)	20 (26.0) (16.6, 37.2)	14 (17.7) (10.0, 27.9)	8 (10.3) (4.5, 19.2)	42 (17.9) (13.3, 23.5)	6 (20.7) (8.0, 39.7)	5 (16.7) (5.6, 34.7)	5 (17.9) (6.1, 36.9)	16 (18.4) (10.9, 28.1)
Mild (grade 1)	22 (28.6)	15 (19.0)	9 (11.5)	46 (19.7)	19 (24.7)	13 (16.5)	6 (7.7)	38 (16.2)	6 (20.7)	5 (16.7)	4 (14.3)	15 (17.2)
Moderate (grade 2)	1 (1.3)	1 (1.3)	3 (3.8)	5 (2.1)	1 (1.3)	1 (1.3)	2 (2.6)	4 (1.7)	0	0	1 (3.6)	1 (1.1)
Severe (grade 3)	0	0	0	0	0	0	0	0	0	0	0	0
Nausea and/or vomiting, any, n (%) (95% CI)[d]	4 (5.2) (1.4, 12.8)	4 (5.1) (1.4, 12.5)	4 (5.1) (1.4, 12.6)	12 (5.1) (2.7, 8.8)	4 (5.2) (1.4, 12.8)	4 (5.1) (1.4, 12.5)	3 (3.8) (0.8, 10.8)	11 (4.7) (2.4, 8.3)	0 (0.0, 11.9)	0 (0.0, 11.6)	1 (3.6) (0.1, 18.3)	1 (1.1) (0.0, 6.2)
Mild (grade 1)	3 (3.9)	1 (1.3)	4 (5.1)	8 (3.4)	3 (3.9)	1 (1.3)	3 (3.8)	7 (3.0)	0	0	1 (3.6)	1 (1.1)
Moderate (grade 2)	1 (1.3)	2 (2.5)	0	3 (1.3)	1 (1.3)	2 (2.5)	0	3 (1.3)	0	0	0	0
Severe (grade 3)	0	1 (1.3)	0	1 (0.4)	0	1 (1.3)	0	1 (0.4)	0	0	0	0
Loss of appetite, any, n (%) (95% CI)[e]	10 (13.0) (6.4, 22.6)	14 (17.7) (10.0, 27.9)	11 (14.1) (7.3, 23.8)	35 (15.0) (10.6, 20.2)	7 (9.1) (3.7, 17.8)	11 (13.9) (7.2, 23.5)	7 (9.0) (3.7, 17.6)	25 (10.7) (7.0, 15.4)	3 (10.3) (2.2, 27.4)	3 (10.0) (2.1, 26.5)	5 (17.9) (6.1, 36.9)	11 (12.6) (6.5, 21.5)
Mild (grade 1)	8 (10.4)	9 (11.4)	7 (9.0)	24 (10.3)	6 (7.8)	6 (7.6)	6 (7.7)	18 (7.7)	2 (6.9)	3 (10.0)	2 (7.1)	7 (8.0)
Moderate (grade 2)	2 (2.6)	5 (6.3)	4 (5.1)	11 (4.7)	1 (1.3)	5 (6.3)	1 (1.3)	7 (3.0)	1 (3.4)	0	3 (10.7)	4 (4.6)
Severe (grade 3)	0	0	0	0	0	0	0	0	0	0	0	0

	After any vaccination				After first vaccination				After second vaccination			
**Season 2**	**Batch 4 (n=77)**	**Batch 5 (n=79)**	**Batch 6 (n=78)**	**Total (n=234)**	**Batch 4 (n=77)**	**Batch 5 (n=79)**	**Batch 6 (n=78)**	**Total (n=234)**	**Batch 4 (n=29)**	**Batch 5 (n=30)**	**Batch 6 (n=28)**	**Total (n=87)**
Irritability, any, n (%) (95% CI)[d]	27 (35.1) (24.5, 46.8)	34 (43.0) (31.9, 54.7)	33 (42.3) (31.2, 54.0)	94 (40.2) (33.8, 46.8)	25 (32.5) (22.2, 44.1)	32 (40.5) (29.6, 52.1)	29 (37.2) (26.5, 48.9)	86 (36.8) (30.6, 43.3)	9 (31.0) (15.3, 50.8)	4 (13.3) (3.8, 30.7)	11 (39.3) (21.5, 59.4)	24 (27.6) (18.5, 38.2)
Mild (grade 1)	12 (15.6)	23 (29.1)	22 (28.2)	57 (24.4)	14 (18.2)	22 (27.8)	23 (29.5)	59 (25.2)	2 (6.9)	2 (6.7)	4 (14.3)	8 (9.2)
Moderate (grade 2)	15 (19.5)	11 (13.9)	10 (12.8)	36 (15.4)	11 (14.3)	10 (12.7)	6 (7.7)	27 (11.5)	7 (24.1)	2 (6.7)	6 (21.4)	15 (17.2)
Severe (grade 3)	0	0	1 (1.3)	1 (0.4)	0	0	0	0	0	0	1 (3.6)	1 (1.1)

CI, confidence interval.

[a]Grade 1: minor reaction on touch; grade 2: cried/protested on touch; grade 3: cried when limb was moved/spontaneously painful.

[b]Grade 1: <10 mm; grade 2: ≥10 to ≤30 mm; grade 3; ≥30 mm.

[c]Axillary measurement: grade 1: ≥37.5 to <38.0°C; grade 2; ≥38.0 to <38.5°C; grade 3; ≥38.5°C. Oral measurement: grade 1: ≥38.0 to <38.5°C; grade 2: ≥38.5 to <39.0°C; grade 3: ≥39.0°C.

[d]Grade 1: easily tolerated, causing minimal discomfort and does not interfere with daily activities; grade 2: sufficiently discomforting to interfere with daily activities; grade 3: prevents normal everyday activities or requires significant medical intervention.

Table S2. Solicited local and systemic adverse reactions experienced after any vaccination and after the first and second vaccinations in participants aged 3 to <9 years, Day 1–7 (solicited safety population)

	After any vaccination				After first vaccination				After second vaccination			
**Season 1**	**Batch 1 (n=97)**	**Batch 2 (n=101)**	**Batch 3 (n=100)**	**Total (n=298)**	**Batch 1 (n=97)**	**Batch 2 (n=100)**	**Batch 3 (n=100)**	**Total (n=297)**	**Batch 1 (n=58)**	**Batch 2 (n=49)**	**Batch 3 (n=59)**	**Total (n=166)**
**Local reactions, n (%)**												
Pain at injection site, any, n (%) (95% CI)[a]	53 (54.6) (44.2, 64.8)	60 (59.4) (49.2, 69.1)	64 (64.0) (53.8, 73.4)	177 (59.4) (53.6, 65.0)	39 (40.2) (30.4, 50.7)	53 (53.0) (42.8, 63.1)	51 (51.0) (40.8, 61.1)	143 (48.1) (42.3, 54.0)	29 (50.0) (36.6, 63.4)	24 (49.0) (34.4, 63.7)	27 (45.8) (32.7, 59.2)	80 (48.2) (40.4, 56.1)
Mild (grade 1)	46 (47.4)	55 (54.5)	53 (53.0)	154 (51.7)	34 (35.1)	48 (48.0)	42 (42.0)	124 (41.8)	26 (44.8)	24 (49.0)	25 (42.4)	75 (45.2)
Moderate (grade 2)	6 (6.2)	5 (5.0)	11 (11.0)	22 (7.4)	4 (4.1)	5 (5.0)	9 (9.0)	18 (6.1)	3 (5.2)	0	2 (3.4)	5 (3.0)
Severe (grade 3)	1 (1.0)	0	0	1 (0.3)	1 (1.0)	0	0	1 (0.3)	0	0	0	0
Erythema at injection site, any, n (%) (95% CI)[b]	17 (17.5) (10.6, 26.6)	27 (26.7) (18.4, 36.5)	21 (21.0) (13.5, 30.3)	65 (21.8) (17.3, 26.9)	14 (14.4) (8.1, 23.0)	24 (24.0) (16.0, 33.6)	19 (19.0) (11.8, 28.1)	57 (19.2) (14.9, 24.1)	3 (5.2) (1.1, 14.4)	5 (10.2) (3.4, 22.2)	6 (10.2) (3.8, 20.8)	14 (8.4) (4.7, 13.7)
Mild (grade 1)	7 (7.2)	14 (13.9)	12 (12.0)	33 (11.1)	5 (5.2)	13 (13.0)	10 (10.0)	28 (9.4)	2 (3.4)	2 (4.1)	5 (8.5)	9 (5.4)
Moderate (grade 2)	7 (7.2)	9 (8.9)	5 (5.0)	21 (7.0)	7 (7.2)	8 (8.0)	5 (5.0)	20 (6.7)	0	2 (4.1)	0	2 (1.2)
Severe (grade 3)	3 (3.1)	4 (4.0)	4 (4.0)	11 (3.7)	2 (2.1)	3 (3.0)	4 (4.0)	9 (3.0)	1 (1.7)	1 (2.0)	1 (1.7)	3 (1.8)
Induration/swelling at injection site, any n (%) (95% CI)[b]	10 (10.3) (5.1, 18.1)	11 (10.9) (5.6, 18.7)	13 (13.0) (7.1, 21.2)	34 (11.4) (8.0, 15.6)	10 (10.3) (5.1, 18.1)	8 (8.0) (3.5, 15.2)	13 (13.0) (7.1, 21.2)	31 (10.4) (7.2, 14.5)	0 (0.0, 6.2)	5 (10.2) (3.4, 22.2)	1 (1.7) (0.0, 9.1)	6 (3.6) (1.3, 7.7)
Mild (grade 1)	6 (6.2)	4 (4.0)	6 (6.0)	16 (5.4)	6 (6.2)	3 (3.0)	6 (6.0)	15 (5.1)	0	2 (4.1)	0	2 (1.2)
Moderate (grade 2)	4 (4.1)	4 (4.0)	4 (4.0)	12 (4.0)	4 (4.1)	3 (3.0)	4 (4.0)	11 (3.7)	0	2 (4.1)	0	2 (1.2)
Severe (grade 3)	0	3 (3.0)	3 (3.0)	6 (2.0)	0	2 (2.0)	3 (3.0)	5 (1.7)	0	1 (2.0)	1 (1.7)	2 (1.2)
**Systemic reactions, n (%)**												
Fever, any, n (%) (95% CI)[c]	8 (8.2) (3.6, 15.6)	4 (4.0) (1.1, 9.8)	11 (11.0) (5.6, 18.8)	23 (7.7) (5.0, 11.4)	7 (7.2) (3.0, 14.3)	3 (3.0) (0.6, 8.5)	8 (8.0) (3.5, 15.2)	18 (6.1) (3.6, 9.4)	2 (3.4) (0.4, 11.9)	1 (2.0) (0.1, 10.9)	3 (5.1) (1.1, 14.1)	6 (3.6) (1.3, 7.7)
Mild (grade 1)	4 (4.1)	2 (2.0)	6 (6.0)	12 (4.0)	3 (3.1) 3	2 (2.0)	5 (5.0)	10 (3.4)	2 (3.4) 2	0	1 (1.7)	3 (1.8)
Moderate (grade 2)	2 (2.1)	0	2 (2.0)	4 (1.3)	2 (2.1)	0	1 (1.0)	3 (1.0)	0	0	1 (1.7)	1 (0.6)
Severe (grade 3)	2 (2.1)	2 (2.0)	3 (3.0)	7 (2.3)	2 (2.1)	1 (1.0)	2 (2.0)	5 (1.7)	0	1 (2.0)	1 (1.7)	2 (1.2)
Fever ≥38°C	6 (6.2)	3 (3.0)	7 (7.0)	16 (5.4)	6 (6.2)	2 (2.0)	5 (5.0)	13 (4.4)	1 (1.7)	1 (2.0)	2 (3.4)	4 (2.4)
Fever ≥39°C	1 (1.0)	1 (1.0)	2 (2.0)	4 (1.3)	1 (1.0)	1 (1.0)	1 (1.0)	3 (1.0)	0	0	1 (1.7)	1 (0.6)
Diarrhoea, any, n (%) (95% CI)[d]	13 (13.4) (7.3, 21.8)	8 (7.9) (3.5,15.0)	15 (15.0) (8.6, 23.5)	36 (12.1) (8.6,16.3)	10 (10.3) (5.1, 18.1)	7 (7.0) (2.9, 13.9)	11 (11.0) (5.6, 18.8)	28 (9.4) (6.4, 13.3)	5 (8.6) (2.9, 19.0)	1 (2.0) (0.1, 10.9)	5 (8.5) (2.8, 18.7)	11 (6.6) (3.4, 11.5)
Mild (grade 1)	10 (10.3)	7 (6.9)	11 (11.0)	28 (9.4)	7 (7.2)	6 (6.0)	9 (9.0)	22 (7.4)	4 (6.9)	1 (2.0)	3 (5.1)	8 (4.8)
Moderate (grade 2)	3 (3.1)	1 (1.0)	3 (3.0)	7 (2.3)	3 (3.1)	1 (1.0)	2 (2.0)	6 (2.0)	1 (1.7)	0	1 (1.7)	2 (1.2)

	After any vaccination				After first vaccination				After second vaccination			
**Season 1**	**Batch 1 (n=97)**	**Batch 2 (n=101)**	**Batch 3 (n=100)**	**Total (n=298)**	**Batch 1 (n=97)**	**Batch 2 (n=100)**	**Batch 3 (n=100)**	**Total (n=297)**	**Batch 1 (n=58)**	**Batch 2 (n=49)**	**Batch 3 (n=59)**	**Total (n=166)**
Severe (grade 3)	0	0	1 (1.0)	1 (0.3)	0	0	0	0	0	0	1 (1.7)	1 (0.6)
Nausea and/or vomiting, any, n (%) (95% CI)[d]	11 (11.3)<br>(5.8,19.4)	6 (5.9)<br>(2.2,12.5)	13 (13.0)<br>(7.1, 21.2)	30 (10.1)<br>(6.9,14.1)	5 (5.2)<br>(1.7, 11.6)	5 (5.0)<br>(1.6, 11.3)	8 (8.0)<br>(3.5, 15.2)	18 (6.1)<br>(3.6, 9.4)	6 (10.3)<br>(3.9, 21.2)	1 (2.0)<br>(0.1, 10.9)	6 (10.2)<br>(3.8, 20.8)	13 (7.8)<br>(4.2, 13.0)
Mild (grade 1)	6 (6.2)	2 (2.0)	9 (9.0)	17 (5.7)	3 (3.1)	2 (2.0)	7 (7.0)	12 (4.0)	3 (5.2)	0	3 (5.1) 3	6 (3.6)
Moderate (grade 2)	5 (5.2)	3 (3.0)	4 (4.0)	12 (4.0)	2 (2.1)	2 (2.0)	1 (1.0)	5 (1.7)	3 (5.2)	1 (2.0)	3 (5.1)	7 (4.2)
Severe (grade 3)	0	1 (1.0)	0	1 (0.3)	0	1 (1.0)	0	1 (0.3)	0	0	0	0
Headache, any, n (%) (95% CI)[d]	16 (16.5)<br>(9.7, 25.4)	13 (12.9)<br>(7.0, 21.0)	18 (18.0)<br>(11.0, 26.9)	47 (15.8)<br>(11.8, 20.4)	12 (12.4)<br>(6.6, 20.6)	11 (11.0)<br>(5.6, 18.8)	14 (14.0)<br>(7.9, 22.4)	37 (12.5)<br>(8.9, 16.8)	4 (6.9)<br>(1.9, 16.7)	4 (8.2)<br>(2.3, 19.6)	5 (8.5)<br>(2.8, 18.7)	13 (7.8)<br>(4.2, 13.0)
Mild (grade 1)	9 (9.3)	10 (9.9)	14 (14.0)	33 (11.1)	7 (7.2)	9 (9.0)	10 (10.0)	26 (8.8)	2 (3.4)	3 (6.1)	5 (8.5)	10 (6.0)
Moderate (grade 2)	6 (6.2)	2 (2.0)	4 (4.0)	12 (4.0)	4 (4.1)	1 (1.0)	4 (4.0) 4	9 (3.0)	2 (3.4)	1 (2.0)	0	3 (1.8)
Severe (grade 3)	1 (1.0)	1 (1.0)	0	2 (0.7)	1 (1.0)	1 (1.0)	0	2 (0.7)	0	0	0	0
Myalgia, any, n (%) (95% CI)[d]	15 (15.5)<br>(8.9, 24.2)	15 (14.9)<br>(8.6, 23.3)	17 (17.0)<br>(10.2, 25.8)	47 (15.8)<br>(11.8,20.4)	14 (14.4)<br>(8.1, 23.0)	12 (12.0)<br>(6.4, 20.0)	14 (14.0)<br>(7.9, 22.4)	40 (13.5)<br>(9.8, 17.9)	3 (5.2)<br>(1.1, 14.4)	7 (14.3)<br>(5.9, 27.2)	7 (11.9)<br>(4.9, 22.9)	17 (10.2)<br>(6.1, 15.9)
Mild (grade 1)	11 (11.3)	13 (12.9)	11 (11.0)	35 (11.7)	10 (10.3)	11 (11.0)	8 (8.0)	29 (9.8)	3 (5.2)	5 (10.2)	7 (11.9)	15 (9.0)
Moderate (grade 2)	3 (3.1)	2 (2.0)	5 (5.0)	10 (3.4)	3 (3.1)	1 (1.0)	5 (5.0)	9 (3.0)	0	2 (4.1)	0	2 (1.2)
Severe (grade 3)	1 (1.0)	0	1 (1.0)	2 (0.7)	1 (1.0)	0	1 (1.0)	2 (0.7)	0	0	0	0
Malaise and fatigue, any, n (%) (95% CI)[d]	29 (29.9)<br>(21.0, 40.0)	23 (22.8)<br>(15.0,32.2)	26 (26.0)<br>(17.7, 35.7)	78 (26.2)<br>(21.3, 31.6)	23 (23.7)<br>(15.7, 33.4)	21 (21.0)<br>(13.5, 30.3)	20 (20.0)<br>(12.7, 29.2)	64 (21.5)<br>(17.0, 26.7)	9 (15.5)<br>(7.3, 27.4)	3 (6.1)<br>(1.3, 16.9)	8 (13.6)<br>(6.0, 25.0)	20 (12.0)<br>(7.5, 18.0)
Mild (grade 1)	19 (19.6)	15 (14.9)	17 (17.0)	51 (17.1)	15 (15.5)	14 (14.0)	13 (13.0)	42 (14.1)	7 (12.1)	2 (4.1)	6 (10.2)	15 (9.0)
Moderate (grade 2)	9 (9.3)	7 (6.9)	9 (9.0)	25 (8.4)	7 (7.2)	6 (6.0)	7 (7.0)	20 (6.7)	2 (3.4)	1 (2.0)	2 (3.4)	5 (3.0)
Severe (grade 3)	1 (1.0)	1 (1.0)	0	2 (0.7)	1 (1.0)	1 (1.0)	0	2 (0.7)	0	0	0	0

	After any vaccination				After first vaccination				After second vaccination			
**Season 2**	**Batch 4 (n=107)**	**Batch 5 (n=109)**	**Batch 6 (n=109)**	**Total (n=325)**	**Batch 4 (n=107)**	**Batch 5 (n=109)**	**Batch 6 (n=109)**	**Total (n=325)**	**Batch 4 (n=19)**	**Batch 5 (n=20)**	**Batch 6 (n=24)**	**Total (n=63)**
**Local reactions, n (%)**												
Pain at injection site, any, n (%) (95% CI)[a]	54 (50.5)<br>(40.6, 60.3)	57 (52.3)<br>(42.5, 61.9)	63 (57.8)<br>(48.0, 67.2)	174 (53.5)<br>(48.0, 59.1)	48 (44.9)<br>(35.2, 54.8)	54 (49.5)<br>(39.8, 59.3)	62 (56.9)<br>(47.0, 66.3)	164 (50.5)<br>(44.9, 56.0)	11 (57.9)<br>(33.5, 79.7)	9 (45.0)<br>(23.1, 68.5)	8 (33.3)<br>(15.6, 55.3)	28 (44.4)<br>(31.9, 57.5)
Mild (grade 1)	45 (42.1)	51 (46.8)	53 (48.6)	149 (45.8)	39 (36.4)	48 (44.0)	52 (47.7)	139 (42.8)	10 (52.6)	9 (45.0)	8 (33.3)	27 (42.9)
Moderate (grade 2)	9 (8.4)	6 (5.5)	10 (9.2)	25 (7.7)	9 (8.4)	6 (5.5)	10 (9.2)	25 (7.7)	1 (5.3)	0	0	1 (1.6)

	After any vaccination				After first vaccination				After second vaccination			
**Season 2**	**Batch 4 (n=107)**	**Batch 5 (n=109)**	**Batch 6 (n=109)**	**Total (n=325)**	**Batch 4 (n=107)**	**Batch 5 (n=109)**	**Batch 6 (n=109)**	**Total (n=325)**	**Batch 4 (n=19)**	**Batch 5 (n=20)**	**Batch 6 (n=24)**	**Total (n=63)**
Severe (grade 3)	0	0	0	0	0	0	0	0	0	0	0	0
Erythema at injection site, any, n (%) (95% CI)[b]	21 (19.6) (12.6, 28.4)	21 (19.3) (12.3, 27.9)	18 (16.5) (10.1, 24.8)	60 (18.5) (14.4, 23.1)	21 (19.6) (12.6, 28.4)	21 (19.3) (12.3, 27.9)	18 (16.5) (10.1, 24.8)	60 (18.5) (14.4, 23.1)	2 (10.5) (1.3, 33.1)	1 (5.0) (0.1, 24.9)	0 (0.0, 14.2)	3 (4.8) (1.0, 13.3)
Mild (grade 1)	13(12.1)	14 (12.8)	12 (11.0)	39 (12.0)	14 (13.1)	14 (12.8)	12 (11.0)	40 (12.3)	1 (5.3)	0	0	1 (1.6)
Moderate (grade 2)	6 (5.6)	6 (5.5)	2 (1.8)	14 (4.3)	5 (4.7)	6 (5.5)	2 (1.8)	13 (4.0)	1 (5.3)	1 (5.0)	0	2 (3.2)
Severe (grade 3)	2 (1.9)	1 (0.9)	4 (3.7)	7 (2.2)	2 (1.9)	1 (0.9)	4 (3.7)	7 (2.2)	0	0	0	0
Induration/swelling at injection site, any n (%) (95% CI)[b]	10 (9.3) (4.6, 16.5)	10 (9.2) (4.5, 16.2)	15 (13.8) (7.9, 21.7)	35 (10.8) (7.6, 14.7)	9 (8.4) (3.9, 15.4)	9 (8.3) (3.8, 15.1)	15 (13.8) (7.9, 21.7)	33 (10.2) (7.1, 14.0)	1 (5.3) (0.1, 26.0)	1 (5.0) (0.1, 24.9)	0 (0.0, 14.2)	2 (3.2) (0.4, 11.0)
Mild (grade 1)	4 (3.7)	3 (2.8)	8 (7.3)	15 (4.6)	4 (3.7)	3 (2.8)	8 (7.3)	15 (4.6)	0	0	0	0
Moderate (grade 2)	5 (4.7)	7 (6.4)	4 (3.7)	16 (4.9)	4 (3.7)	6 (5.5)	4 (3.7)	14 (4.3)	1 (5.3)	1 (5.0)	0	2 (3.2)
Severe (grade 3)	1 (0.9)	0	3 (2.8)	4 (1.2)	1 (0.9)	0	3 (2.8)	4 (1.2)	0	0	0	0
**Systemic reactions, n (%)**												
Fever, any, n (%) (95% CI)[c]	2 (1.9) (0.2, 6.6)	3 (2.8) (0.6, 7.8)	2 (1.8) (0.2, 6.5)	7 (2.2) (0.9, 4.4)	2 (1.9) (0.2, 6.6)	2 (1.8) (0.2, 6.5)	2 (1.8) (0.2, 6.5)	6 (1.8) (0.7, 4.0)	0 (0.0, 17.6)	1 (5.0) (0.1, 24.9)	0 (0.0, 14.2)	1 (1.6) (0.0, 8.5)
Mild (grade 1)	1 (0.9)	2 (1.8)	0	3 (0.9)	1 (0.9)	1 (0.9)	0	2 (0.6)	0	1 (5.0)	0	1 (1.6)
Moderate (grade 2)	0	1 (0.9)	0	1 (0.3)	0	1 (0.9)	0	1 (0.3)	0	0	0	0
Severe (grade 3)	1 (0.9)	0	2 (1.8)	3 (0.9)	1 (0.9)	0	2 (1.8)	3 (0.9)	0	0	0	0
Fever ≥38°C	2 (1.9)	1 (0.9)	2 (1.8)	5 (1.5)	2 (1.9)	1 (0.9)	2 (1.8)	5 (1.5)	0	0	0	0
Fever ≥39°C	0	0	2 (1.8)	2 (0.6)	0	0	2 (1.8)	2 (0.6)	0	0	0	0
Diarrhoea, any, n (%) (95% CI)[d]	7 (6.5) (2.7, 13.0)	6 (5.5) (2.0, 11.6)	11 (10.1) (5.1, 17.3)	24 (7.4) (4.8, 10.8)	7 (6.5) (2.7, 13.0)	5 (4.6) (1.5, 10.4)	8 (7.3) (3.2, 14.0)	20 (6.2) (3.8, 9.3)	0 (0.0, 17.6)	1 (5.0) (0.1, 24.9)	3 (12.5) (2.7, 32.4)	4 (6.3) (1.8, 15.5)
Mild (grade 1)	6 (5.6)	6 (5.5)	9 (8.3)	21 (6.5)	6 (5.6)	5 (4.6)	6 (5.5)	17 (5.2)	0	1 (5.0)	3 (12.5)	4 (6.3)
Moderate (grade 2)	1 (0.9)	0	2 (1.8)	3 (0.9)	1 (0.9)	0	2 (1.8)	3 (0.9)	0	0	0	0
Severe (grade 3)	0	0	0	0	0	0	0	0	0	0	0	0
Nausea and/or vomiting, any, n (%) (95% CI)[d]	5 (4.7) (1.5, 10.6)	4 (3.7) (1.0, 9.1)	7 (6.4) (2.6, 12.8)	16 (4.9) (2.8, 7.9)	5 (4.7) (1.5, 10.6)	3 (2.8) (0.6, 7.8)	6 (5.5) (2.0, 11.6)	14 (4.3) (2.4, 7.1)	0 (0.0, 17.6)	1 (5.0) (0.1, 24.9)	1 (4.2) (0.1, 21.1)	2 (3.2) (0.4, 11.0)
Mild (grade 1)	1 (0.9)	3 (2.8)	6 (5.5)	10 (3.1)	1 (0.9)	2 (1.8)	5 (4.6)	8 (2.5)	0	1 (5.0)	1 (4.2)	2 (3.2)
Moderate (grade 2)	2 (1.9)	1 (0.9)	1 (0.9)	4 (1.2)	2 (1.9)	1 (0.9)	1 (0.9)	4 (1.2)	0	0	0	0
Severe (grade 3)	2 (1.9)	0	0	2 (0.6)	2 (1.9)	0	0	2 (0.6)	0	0	0	0

	After any vaccination				After first vaccination				After second vaccination			
**Season 2**	**Batch 4 (n=107)**	**Batch 5 (n=109)**	**Batch 6 (n=109)**	**Total (n=325)**	**Batch 4 (n=107)**	**Batch 5 (n=109)**	**Batch 6 (n=109)**	**Total (n=325)**	**Batch 4 (n=19)**	**Batch 5 (n=20)**	**Batch 6 (n=24)**	**Total (n=63)**
Headache, any, n (%) (95% CI)[d]	11 (10.3) (5.2, 17.7)	14 (12.8) (7.2, 20.6)	12 (11.0) (5.8, 18.4)	37 (11.4) (8.1, 15.3)	9 (8.4) (3.9, 15.4)	13 (11.9) (6.5, 19.5)	10 (9.2) (4.5, 16.2)	32 (9.8) (6.8, 13.6)	4 (21.1) (6.1, 45.6)	2 (10.0) (1.2, 31.7)	2 (8.3) (1.0, 27.0)	8 (12.7) (5.6, 23.5)
Mild (grade 1)	7 (6.5)	11 (10.1)	9 (8.3)	27 (8.3)	7 (6.5)	11 (10.1)	8 (7.3)	26 (8.0)	2 (10.5)	1 (5.0)	1 (4.2)	4 (6.3)
Moderate (grade 2)	3 (2.8)	3 (2.8)	3 (2.8)	9 (2.8)	1 (0.9)	2 (1.8)	2 (1.8)	5 (1.5)	2 (10.5)	1 (5.0)	1 (4.2)	4 (6.3)
Severe (grade 3)	1 (0.9)	0	0	1 (0.3)	1 (0.9)	0	0	1 (0.3)	0	0	0	0
Myalgia, any, n (%) (95% CI)[d]	16 (15.0) (8.8, 23.1)	10 (9.2) (4.5, 16.2)	16 (14.7) (8.6, 22.7)	42 (12.9) (9.5, 17.1)	14 (13.1) (7.3, 21.0)	10 (9.2) (4.5, 16.2)	14 (12.8) (7.2, 20.6)	38 (11.7) (8.4, 15.7)	2 (10.5) (1.3, 33.1)	0 (0.0, 16.8)	2 (8.3) (1.0, 27.0)	4 (6.3) (1.8, 15.5)
Mild (grade 1)	14 (13.1)	9 (8.3)	14 (12.8)	37 (11.4)	13 (12.1)	9 (8.3)	12 (11.0)	34 (10.5)	1 (5.3)	0	2 (8.3)	3 (4.8)
Moderate (grade 2)	2 (1.9)	1 (0.9)	2 (1.8)	5 (1.5)	1 (0.9)	1 (0.9)	2 (1.8)	4 (1.2)	1 (5.3)	0	0	1 (1.6)
Severe (grade 3)	0	0	0	0	0	0	0	0	0	0	0	0
Malaise and fatigue, any, n (%) (95% CI)[d]	13 (12.1) (6.6, 19.9)	16 (14.7) (8.6, 22.7)	10 (9.2) (4.5, 16.2)	39 (12.0) (8.7, 16.0)	10 (9.3) (4.6, 16.5)	16 (14.7) (8.6, 22.7)	9 (8.3) (3.8, 15.1)	35 (10.8) (7.6, 14.7)	3 (15.8) (3.4, 39.6)	0 (0.0, 16.8)	1 (4.2) (0.1, 21.1)	4 (6.3) (1.8, 15.5)
Mild (grade 1)	5 (4.7)	9 (8.3)	7 (6.4)	21 (6.5)	4 (3.7)	9 (8.3)	6 (5.5)	19 (5.8)	1 (5.3)	0	1 (4.2)	2 (3.2)
Moderate (grade 2)	6 (5.6)	7 (6.4)	3 (2.8)	16 (4.9)	4 (3.7)	7 (6.4)	3 (2.8)	14 (4.3)	2 (10.5)	0	0	2 (3.2)
Severe (grade 3)	2 (1.9)	0	0	2 (0.6)	2 (1.9)	0	0	2 (0.6)	0	0	0	0

CI, confidence interval.

[a]Grade 1: does not interfere with daily activities; grade 2: interferes with daily activities; grade 3; prevents daily activities.

[b]Grade 1: <10 mm; grade 2: ≥10 to ≤30 mm; grade 3; ≥30 mm.

[c]Axillary measurement: grade 1: ≥37.5 to <38.0°C; grade 2; ≥38.0 to <38.5°C; grade 3; ≥38.5°C. Oral measurement: grade 1: ≥38.0 to <38.5°C; grade 2: ≥38.5 to <39.0°C; grade 3: ≥39.0°C.

[d]Grade 1: easily tolerated, causing minimal discomfort and does not interfere with daily activities; grade 2: sufficiently discomforting to interfere with daily activities; grade 3: prevents normal everyday activities or requires significant medical intervention.

Table S3. Unsolicited AEs occurring in ≥1% of participants by system organ class and preferred term after any vaccination, Day 1–7 (safety population)

**n (%)**	**Age 6 months to <3 years**				**Age 3 to <9 years**			
**Season 1**	**Batch 1 (n=64)**	**Batch 2 (n=57)**	**Batch 3 (n=60)**	**Total (n=181)**	**Batch 1 (n=97)**	**Batch 2 (n=101)**	**Batch 3 (n=100)**	**Total (n=298)**
**Unsolicited AE**								
Any	11 (17.2)	8 (14.0)	14 (23.3)	33 (18.2)	14 (14.4)	6 (5.9)	12 (12.0)	32 (10.7)
Mild	8 (12.5)	4 (7.0)	9 (15.0)	21 (11.6)	10 (10.3)	4 (4.0)	5 (5.0)	19 (6.4)
Moderate	2 (3.1)	3 (5.3)	5 (8.3)	10 (5.5)	4 (4.1)	2 (2.0)	5 (5.0)	11 (3.7)
Severe	1 (1.6)	1 (1.8)	0	2 (1.1)	0	0	2 (2.0)	2 (0.7)
Any related	1 (1.6)	1 (1.8)	2 (3.3)	4 (2.2)	6 (6.2)	1 (1.0)	1 (1.0)	8 (2.7)
**Eye disorders**								
Eye discharge	0	0	0	0	1 (1.0)	0	0	1 (0.3)
**Infections and infestations**								
Upper respiratory tract infection	1 (1.6)	1 (1.8)	1 (1.7)	3 (1.7)	0	2 (2.0)	1 (1.0)	3 (1.0)
Conjunctivitis	1 (1.6)	1 (1.8)	0	2 (1.1)	0	1 (1.0)	0	1 (0.3)
Hand-foot-and-mouth disease	1 (1.6)	0	1 (1.7)	2 (1.1)	0	0	0	0
Tonsillitis	1 (1.6)	1 (1.8)	0	2 (1.1)	1 (1.0)	0	1 (1.0)	2 (0.7)
Croup infectious	0	0	1 (1.7)	1 (0.6)				
Ear infection	1 (1.6)	0	0	1 (0.6)	1 (1.0)	0	2 (2.0)	3 (1.0)
Otitis media	0	0	1 (1.7)	1 (0.6)	0	0	0	0
Respiratory syncytial virus bronchiolitis	0	1 (1.8)	0	1 (0.6)	0	0	0	0
Roseola	0	0	1 (1.7)	1 (0.6)	0	0	0	0
Viraemia	1 (1.6)	0	0	1 (0.6)	0	0	0	0
Viral upper respiratory tract infection	1 (1.6)	0	0	1 (0.6)	2 (2.1)	0	1 (1.0) 1	3 (1.0)
Influenza	0	0	0	0	1 (1.0)	0	1 (1.0) 1	2 (0.7)
Mycoplasma infection	0	0	0	0	1 (1.0)	0	0	1 (0.3)
Varicella	0	0	0	0	1 (1.0)	0	0	1 (0.3)
Viral infection	0	0	0	0	0	0	1 (1.0)	1 (0.3)
**Gastrointestinal disorders**								
Teething	0	0	4 (6.7)	4 (2.2)	0	0	0	0
Vomiting[a]	0	1 (1.8)	3 (5.0)	4 (2.2)	1 (1.0)	1 (1.0)	0	2 (0.7)
Abdominal pain upper	0	0	0-	-	1 (1.0)	0	1 (1.0)	2 (0.7)
**General disorders and administration site conditions**								
Injection site bruising[a]	1 (1.6)	1 (1.8)	1 (1.7)	3 (1.7)	0	0	0	0
Pyrexia[a]	1 (1.6)	1 (1.8)	0	2 (1.1)	1 (1.0)	1 (1.0)	2 (2.0) 2	4 (1.3)
Chills	0	0	1 (1.7)	1 (0.6)	0	0	0	0
Influenza like illness	1 (1.6)	0	0	1 (0.6)	0	0	0	0
Feeling hot[a]	0	0	0	0	1 (1.0)	0	0	1 (0.3)
Injection site pain[a]	0	0	0	0	0	0	1 (1.0)	1 (0.3)
Injection site swelling[a]	0	0	0	0	1 (1.0)	0	0	1 (0.3)
Pain[a]	0	0	0	0	1 (1.0)	0	0	1 (0.3)
Swelling[a]	0	0	0	0	1 (1.0)	0	0	1 (0.3)
**Injury, poisoning and procedural complications**								
Fall	0	1 (1.8)	0	1 (0.6)	0	0	0	0
Scratch	1 (1.6)	0	0	1 (0.6)	0	0	0	0
Skin laceration	0	1 (1.8)	0	1 (0.6)	0	0	0	0
Concussion	0	0	0	0	0	1 (1.0)	0	1 (0.3)

n (%)	Age 6 months to <3 years				Age 3 to <9 years			
Season 1	Batch 1 (n=64)	Batch 2 (n=57)	Batch 3 (n=60)	Total (n=181)	Batch 1 (n=97)	Batch 2 (n=101)	Batch 3 (n=100)	Total (n=298)
Foot fracture	0	0	0	0	0	0	1 (1.0)	1 (0.3)
**Investigations, n (%)**								
Body temperature increased[a]	0	0	0	0	1 (1.0)	0	0	1 (0.3)
Influenza A virus test positive	0	0	0	0	1 (1.0)	0	0	1 (0.3)
Influenza B virus test positive	0	0	0	0	0	0	1 (1.0)	1 (0.3)
**Respiratory, thoracic and mediastinal disorders**								
Asthma	0	0	1 (1.7)	1 (0.6)	0	0	0	0
Nasal congestion	1 (1.6)	0	0	1 (0.6)	0	0	0	0
Pharyngeal inflammation	0	0	1 (1.7)	1 (0.6)	0	0	0	0
Cough	0	0	0	0	3 (3.1)	0	1 (1.0)	4 (1.3)
Rhinorrhoea[a]	0	0	0	0	1 (1.0)	0	1 (1.0)	2 (0.7)
**Skin and subcutaneous tissue disorders**								
Eczema	0	0	1 (1.7)	1 (0.6)	0	0	0	0
Erythema[a]	0	1 (1.8)	0	1 (0.6)	1 (1.0)	0	0	1 (0.3)
Rash	0	0	0	0	1 (1.0)	0	0	1 (0.3)
**Nervous system disorders**								
Headache	0	1 (1.8)	0	1 (0.6)	0	0	0	0
Lethargy[a]	0	0	0	0	1 (1.0)	0	1 (1.0)	2 (0.7)
**Psychiatric disorders**								
Irritability[a]	0	0	1 (1.7)	1 (0.6)	0	0	1 (1.0)	1 (0.3)

n (%)	6 Months to <3 Years				3 to <9 Years			
Season 2	Batch 4 (n=77)	Batch 5 (n=79)	Batch 6 (n=78)	Total (n=234)	Batch 4 (n=107)	Batch 5 (n=110)	Batch 6 (n=109)	Total (n=326)
**Unsolicited AE**								
Any	10 (13.0)	11 (13.9)	13 (16.7)	34 (14.5)	5 (4.7)	9 (8.2)	5 (4.6)	19 (5.8)
Mild	7 (9.1)	5 (6.3)	8 (10.3)	20 (8.5)	4 (3.7)	6 (5.5)	4 (3.7)	14 (4.3)
Moderate	3 (3.9)	5 (6.3)	3 (3.8)	11 (4.7)	1 (0.9)	2 (1.8)	0	3 (0.9)
Severe	0	1 (1.3)	2 (2.6)	3 (1.3)	0	1 (0.9)	1 (0.9)	2 (0.6)
Any related	2 (2.6)	2 (2.5)	2 (2.6)	6 (2.6)	0	4 (3.6)	1 (0.9)	5 (1.5)
**Infections and infestations**								
Upper respiratory tract infection	0	2 (2.5)	3 (3.8)	5 (2.1)	0	1 (0.9)	1 (0.9)	2 (0.6)
Viral infection	2 (2.6)	0	0	2 (0.9)	0	0	0	0
Adenovirus infection	0	0	1 (1.3)	1 (0.4)	0	0	0	0
Croup infectious	0	1 (1.3)	0	1 (0.4)	0	0	0	0
Ear infection	0	0	1 (1.3)	1 (0.4)	0	0	1 (0.9)	1 (0.3)
Gastroenteritis	0	1 (1.3)	0	1 (0.4)	0	0	0	0
Rhinitis	0	0	1 (1.3)	1 (0.4)	0	0	0	0
Viral upper respiratory tract infection	0	0	1 (1.3)	1 (0.4)	0	0	0	0
**Gastrointestinal disorders**								
Teething	2 (2.6)	1 (1.3)	2 (2.6)	5 (2.1)	0	0	0	0
Constipation	1 (1.3)	0	0	1 (0.4)	0	0	0	0
Diarrhoea[a]	0	0	1 (1.3)	1 (0.4)	0	1 (0.9)	0	1 (0.3)
Mouth ulceration[a]	0	1 (1.3)	0	1 (0.4)	0	0	0	0
Oesophagitis	0	1 (1.3)	0	1 (0.4)	0	0	0	0

n (%)	6 Months to <3 Years				3 to <9 Years			
Season 2	Batch 4 (n=77)	Batch 5 (n=79)	Batch 6 (n=78)	Total (n=234)	Batch 4 (n=107)	Batch 5 (n=110)	Batch 6 (n=109)	Total (n=326)
**General disorders and administration site conditions**								
Injection site bruising[a]	2 (2.6)	0	0	2 (0.9)	0	0	0	0
Injection site erythema[a]	0	0	1 (1.3)	1 (0.4)	0	0	1 (0.9)	1 (0.3)
Pyrexia[a]	0	1 (1.3)	0	1 (0.4)	0	1 (0.9)	0	1 (0.3)
Injection site pain[a]	0	0	0	0	0	2 (1.8)	0	2 (0.6)
**Injury, poisoning and procedural complications**								
Head injury	1 (1.3)	1 (1.3)	0	2 (0.9)	0	0	0	0
Eye contusion	1 (1.3)	0	0	1 (0.4)	0	0	0	0
Fall	1 (1.3)	0	0	1 (0.4)	0	0	0	0
**Ear and labyrinth disorders**								
Ear haemorrhage	0	1 (1.3)	0	1 (0.4)	0	0	0	0
**Respiratory, thoracic and mediastinal disorders**								
Rhinorrhoea[a]	2 (2.6)	0	2 (2.6)	4 (1.7)	1 (0.9)	1 (0.9)	0	2 (0.6)
Cough	1 (1.3)	0	0	1 (0.4)	1 (0.9)	0	1 (0.9)	2 (0.6)
Oropharyngeal pain	0	0	0	0	2 (1.9)	1 (0.9)	0	3 (0.9)
**Skin and subcutaneous tissue disorders**								
Dermatitis diaper	0	1 (1.3)	0	1 (0.4)	0	0	0	0
Eczema	0	0	1 (1.3)	1 (0.4)	0	0	0	0
**Psychiatric disorders**								
Irritability[a]	1 (1.3)	0	1 (1.3)	2 (0.9)	0	0	0	0

AE, adverse event

[a]Related AE.
